# Genomic insights into nitrofurantoin resistance mechanisms and epidemiology in clinical Enterobacteriaceae

**DOI:** 10.4155/fsoa-2017-0156

**Published:** 2018-02-27

**Authors:** John Osei Sekyere

**Affiliations:** 1 Department of Pharmaceutics, Faculty of Pharmacy & Pharmaceutical Sciences, KNUST, Kumasi, Ghana; 2 Discipline of Pharmaceutical Sciences, School of Health Sciences, University of KwaZulu-Natal, Durban, South Africa

**Keywords:** antibiotic resistance mechanisms, comparative genomics, efflux, Enterobacteriaceae, nitrofurantoin, South Africa

## Abstract

**Aim:**

Multidrug-resistant enterobacteria are highly associated with invasive devices and intensive care units. Increasing resistance to carbapenems is leading to the use of older and neglected antibiotics such as nitrofurantoin (NFT). The genomics of NFT resistance was investigated.

**Results & conclusion:**

High-level resistance to NFT (minimum inhibitory concentration ≥128–512 mg/l) was recorded in 31/36 isolates (89.6%), many of which were from intensive care units (n = 20), urine (n = 17) or invasive procedures (n = 10). Efflux pump inhibitors had little effect on NFT's minimum inhibitory concentrations albeit *oqxAB* was prevalent in most isolates (n = 32). Various species- and clone-specific mutations mediating high-level NFT resistance were detected in nfsA, nfsB and ribE proteins through comparative genomics. Global phylogenomics showed local and independent emergence of NFT resistance in Enterobacteriaceae. NFT stewardship is advised.

## Lay abstract

Nitrofurantoin (NFT) is an important antibiotic indicated for uncomplicated urinary tract infections. Resistance to NFT is slow and uncommon, making it an important drug for treating urinary tract infections resistant to common and last-resort antibiotics such as cephalosporins, fluoroquinolones, aminoglycosides and carbapenems. Increasing resistance to most antibiotics among uropathogens makes NFT a key choice for clinicians. Thus, the high-level NFT resistance in extradrug- and pandrug-resistant uropathogens found in this study is exceptionally worrying as treatment options will be extremely limited. Mutations in *nfsA, nfsB* and *ribE* genes in the same and different strains emerged locally and independently to confer NFT resistance.

The worrying decline in carbapenems’ efficacy as last-line antibiotics for difficult-to-treat Gram-negative bacterial infections is increasing both the quest for novel antibiotics and the reintroduction of older ones into clinical medicine [1–4]. Tigecycline, for instance, is a novel glycylcycline antibiotic with great efficacy against carbapenem-resistant infections while colistin is an old antibiotic with adverse nephrotoxic and neurotoxic effects that has been reintroduced clinically to combat multidrug-resistant (MDR) infections [2,4,5]. Due to the inability of carbapenems alone to salvage fatal carbapenem-resistant bacterial infections, they are either used with tigecycline or colistin in many clinical infectious disease cases [2,4]. As well, other older but more toxic antibiotics such as nitrofurantoin (NFT) and fosfomycin are being reintroduced alongside carbapenems, colistin and tigecycline to combat antibiotic-resistant infections [6,7]. Particularly, fosfomycin and NFT are becoming important for urinary tract infections (UTIs) that are resistant to carbapenems [8].

NFT is a broad-spectrum antibiotic that has been used for the treatment of uncomplicated UTIs since the mid-1950s [9]. In recent years, interest in NFT has been revived as the post-antibiotic era looms, and its efficacy against carbapenem-resistant infections is becoming evident [10,11]. The precise mechanism of action of NFT is not known, albeit it has been shown to damage DNA and inhibit total protein production in *Escherichia coli* by reacting nonspecifically with both ribosomal proteins and rRNA [12]. This is preceded by the activation of NFT by bacterial reductases to highly-reactive electrophilic intermediates; an inverse correlation exists with the reductase activity of the bacteria and its NFT MIC [13].

NFT resistance is mainly mediated by mutations in *nfsA* and/or *nfsB*, both of which encode oxygen-insensitive nitroreductases responsible for high-level nitrofurantoin resistance (NFT-R; median MIC of 96 μg/ml) [13]. These mutations hinder the reduction of NFT, preventing the formation of toxic intermediate compounds. Furthermore, deletion(s) in the *ribE* gene, which encodes lumazine synthase that is needed for riboflavin biosynthesis, has been shown to increase MIC levels in laboratory mutants, although these mutations have so far not been described in clinical isolates. Deletions in ribE thus leads to NFT resistance by inhibiting the synthesis of riboflavin/flavin mononucleotide, an important cofactor of nfsA and nfsB [14,15]. Recently, plasmid-mediated efflux genes, *oqxAB*, have also been associated with clinically relevant levels of NFT-R, implicating the dissemination of these MDR efflux pumps in NFT-R [16].

The resistance mechanisms and evolutionary biology of clinical Enterobacteriaceae isolates are herein described.

## Methods

### Bioinformatic analyses

The isolates and the phenotypic assays used in characterizing their NFT resistance are described in the Supplementary Appendix. All the isolates were highly resistant to fluoroquinolones (MIC: 4–512 mg/l) [17] and 34 were highly resistant to imipenem and meropenem, with most isolates having a meropenem and imipenem MIC of 128–512 mg/l [5]. 31 isolates were resistant to colistin (MIC: 4–256 mg/l) and 30 were highly resistant to tigecycline (MIC: 2–8 mg/l) [5]. All the isolates were resistant to the penicillins and cephalosporins [18], and most were resistant to all the above-listed antibiotics, making the strains MDR and pandrug-resistant.

Raw genome sequence reads of the 36 isolates were downloaded from the SRA website (PRJNA287968) and assembled with SPAdes 3.9 [19]. Chromosomal and plasmid-mediated efflux pump genes, *oqxAB*, which have been implicated in NFT-R were annotated with ResFinder [20] and confirmed with CARD [21] using both raw and assembled reads, respectively. To determine whether the *oqxAB* genes in *Klebsiella pneumoniae* ST101 and ST2017 were chromosomal or plasmid-mediated, *oqxAB* nucleotide sequences were BLASTed against the chromosomes (CP023553.1 and CP023487.1) and plasmid (CP023555.1, CP023489.1, CP023488.1 and CP023554.1) genomes of these strains. The genetic environments of *oqxAB* were searched for IS26 and other insertion sequences or transposons already reported to be associated with *oqxAB* genes using the NCBI Prokaryotic Genome Annotation Pipeline [22,16].

Mutations in the chromosome-borne nfsA, nfsB and ribE proteins implicated in NFT-R were determined using tBLASTn. Briefly, these proteins in wild-type reference strains were respectively aligned to those of the same species within the 36 isolates to identify mutations, truncations, insertions and deletions (Tables 1–3). At least four NFT-susceptible *E. coli* and *K. pneumoniae* genomes were downloaded from PATRIC [23] and NCBI/Genbank to determine amino acid mutations in nfsA, nfsB and ribE. Only single reference strains were used for *Citrobacter freundii* and *Enterobacter* spp. due to the absence of specified NFT-susceptible strains at PATRIC and Genbank. NFT-susceptible reference/type strains that were used for each species were as follows: *K. pneumoniae* ATCC 13883 (Bioproject number PRJNA244567), strain 155 (NXHL01.1 from bioproject number PRJNA 411997), and strains ST234:K062 (NXKX01.1), ST17:K120 (NXKZ01.1), ST15:K125 (NXKA01.1), ST643:K129 (NXKM01.1), ST14:K118 (NXKP01.1) and ST232:K090 (NXJR01.1) of bioproject PRJNA355910 for *K. pneumoniae*; *Enterobacter cloacae* ATCC 13047 (accession number CP001918.1) for all *Enterobacter* spp. except *Enterobacter asburiae*; *E. asburiae* L1 (accession number CP007546.1) for *E. asburiae*; *Enterobacter kobei* strain 35730 (JZYS01000016.1) or *E. cloacae* ATCC 13047 (accession number CP001918.1) for *E. kobei*; *C. freundii* ATCC 8090 = MTCC 1658 (PRJNA177199) for *C. freundii*; *E. coli ATCC 25922* (accession number CP009072.1), strain 5CRE51 (accession number CP021175.1), ST131:E011 (NXKR01.1), ST131:E056 (NXJD01.1), ST73:E053 (NXIR01.1) and ST95:E040 (NXIP01.1; from bioproject number PRJNA355910) for *E. coli*; *Klebsiella michiganensis* KCTC 1686 (accession number CP003218.1) for *K. michiganensis*.

**Table 1.** **Phenotypic and genomic characteristics of nitrofurantoin resistance mechanisms of the *Klebsiella pneumoniae* isolates.**

**Isolate (clone)**	**MIC (mg/l)**	**Year isolated**	**Specimen type (ward)**	**Plasmid-/chromosomal-mediated *oqxA/B***	**Chromosomal mutations**		

					***nsfA***	***nsfB***	***ribE***
***Klebsiella pneumoniae*^†^**							

53_S27 (ST101)	128	2013	Urine (ICU)	*oqxA, oqxB*	R203C	R207L	R58H, D203E

52_S26 (ST101)	128	2013	Pus (surgical)	*oqxA, oqxB*			

47_S22 (ST1478)	128	2013	Arterial line (ICU)	*oqxA, oqxB*	R59Q, T117I, E144A, R180H, E191D	G25S, V167I	R58H, M204V, A206S, V78I

38_S19 (ST101)	256	X^‡^	X (ICU)	*oqxB*	R203C	R207L	R58H, D203E

36_S18 (ST101)	256	2013	Sputum	*oqxB*			

35_S17(ST101)	256	2013	Urine (ICU)	*oqxB*			

34_S16(ST101)	256	2013	Pus swab (Trachea)	*oqxB*			

32_S15(ST101)	256	2013	Catheter tip (ICU)	*oqxB*			

30_S14(ST101)	256	2013	Urine (ICU)	*oqxB*			

29_S13(ST2017)	256	2012	Abdominal swab (ICU)	*oqxB*			

21_S12(ST2017)	128	2012	Urine (ICU)	*oqxA, oqxB*			

20_S11(ST2017)	256	2013	Tracheal fluid (ICU)	*oqxA, oqxB*			

18_S10(ST101)	256	2013	Urine (neuronal)	*oqxB*			

15_S8(ST101)	512	2013	Pus swab (leg) (neuronal)	*oqxB*			

13_S6 (ST2016)	256	X	X (ICU)	*oqxB*			

12_S5(ST101)	256	2013	Blood culture (ICU)	*oqxB*			

3_S2(ST14)	64	X	Urine (surgical)	*oqxB, oqxA*	NM^§^	NM	D203E

I(UNN45_S9) (ST323)	64	X	Urine (ICU)	*oqxB, oqxA*			

J(UNN46_S10(ST101))	512	X	X	*oqxB*	R203C	R207L	R58H, D203E

D(UNN40_S4) (ST101)	256	X	Urine (ICU)	*oqxB, oqxA*			

C(UNN39_S3) (ST101)	256	X	Urine (surgical)	*oqxA, oqxB*			

^†^ *K. pneumoniae* ATCC 13883 (PRJNA244567) was used as a reference strain in the comparative genome analysis

^‡^Missing data.

^§^No unique mutation recorded in the conserved region of the gene.

ICU: Intensive care unit; MIC: Minimum inhibitory concentration; NM: No mutation.

**Table 2.** **Phenotypic and genomic characteristics of nitrofurantoin resistance mechanisms of the *Enterobacter species* isolates.**

**Isolate (clone)**	**MIC (mg/l)**	**Year isolated**	**Specimen type (ward)**	**Plasmid-/chromosomal-mediated *oqxA/B***	**Chromosomal mutations**		

					***nsfA***	***nsfB***	***ribE***
***Enterobacter species* (unless otherwise stated in footnote, species is *cloacae*)^†^**							

65_S32 (ST436)	256	X	CVP tip (ICU)	*oqxB, oqxA*	H16Y, Q186N, N190A	NM	N55H

63_S31^‡^ (ST435)	128	X	Urine (surgical)	*oqxA, oqxB*	N6D, D19N, T55A, Q186R, M201L	A18P, S19A, M90L, E137Q	I26V

55_S28^§^ (ST434)	128	X	ETA (ICU)	*oqxB, oqxA*	R9L, E28T, D32N, K54P, E94Q, L130I, Q186R, Q191H, H198N, M201L, G204D	A19T, D25E, A155G, L157M, L186V	V42I, Q58K, V77A, V125M, P154A, A173S, Q178H

49_S24^¶^ (ST252)	128	X	Urine (ICU)	*oqxB, oqxA*	N6D, T55A, M201L	A18P, S19A, M90L, E137Q, V214L	I26V

43_S20 (ST433)^#^	256	X		*oqxB, oqxA*	R9L, E24D, D32N, K54P, E137Q, E141D, L170I, Q191H, H198N, S213T	P18A, A19S, D25E	D34E, V42I, V125M, Q178H

16_S9^††^ (ST54)	256	X		*oqxA, oqxB*	R9L, E28T, D32N, K54P, E94Q, L130I, Q186R, Q191H, H198N, M201L, G204D.	A19T, D25E, A155G, L157M, L186V	V42I, Q58K, V77A, V125M, P154A, A173S, Q178H

1_S1(ST108)	256	X		*oqxB, oqxA*	E24Q, E28D, A36G, K54P, L170I, N190H, H198N, M201L, G204D	D25E, N109A, T122C, L157M	V42I, T81I

H(UNN44_S8)^‡‡^ (ST145)	128	X	Urine (ICU)	*oqxA, oqxB*	E24Q, E28D, A36G, K54P, E141Q, L170I, N190H, H198N, M201L, G204D	D25E, N109A, T122C, L157M	V42I

F(UNN42_S6) (ST121)^§§^	256	X	Urine (surgical)	*oqxA, oqxB*	E24Q, E28D, A36G, K54P, A112E, L170I, N190H, H198N, M201L, G204D	D25E, N109A, T122C, L157M	V42I, T81I, V82I

A(UNN37_S1)^¶¶^ (ST252)	512	X	Urine (pediatric)	*oqxB, oqxA*	N6D, T55A, M201L	A18P, S19A, M90L, E137Q, V214L	I26V

^†^Unless otherwise stated in footnote, *Enterobacter cloacae* ATCC 13047 (CP001918.1) was used as a reference strain in the comparative genomics.

^‡^ *Enterobacter asburiae*: *E. asburiae* L1 (CP007546.1) was used as a reference strain for the comparative genomics to find amino acid mutations.

^§^ *Enterobacter kobei*: *E. kobei* strain 35730 (JZYS01000016.1) or *E. cloacae* ATCC 13047 (CP001918.1) served as reference strains for amino acid mutations.

^¶^ *E. asburiae*: *E. asburiae* L1 (CP007546.1) was used as a reference strain for the comparative genomics to find amino acid mutations.

^#^ *E. cloacae complex ‘Hoffman cluster IV’*.

^††^ *E. kobei*: *E. kobei* strain 35730 (JZYS01000016.1) or *E. cloacae* ATCC 13047 (CP001918.1) served as reference strains for amino acid mutations.

^‡‡^ *E. cloacae complex ‘Hoffman cluster III’*.

^§§^ *Enterobacter species*.

^¶¶^ *E. asburiae*: *E. asburiae* L1 (CP007546.1) was used as a reference strain for the comparative genomics to find amino acid mutations.

CVP: Central venous puncture; ETA: Endotracheal aspirate; ICU: Intensive care unit; NM: No mutation.

**Table 3.** **Phenotypic and genomic characteristics of nitrofurantoin resistance mechanisms of the *Citrobacter freundii, Escherichia coli* and *Klebsiella michiganensis* isolates.**

**Isolate (clone)**	**MIC (mg/l)**	**Year isolated**	**Specimen type (ward)**	**Plasmid-/chromosomal-mediated *oqxA/B***	**Chromosomal mutations**		

					***nsfA***	***nsfB***	***ribE***
***C. freundii*^†^**							

51_S25 (NK^‡^)	64	X	Sputum (ICU)	–	Insertion of LGLAEQLLLGVVDTAMI between positions 94 and 95, D185N, T186S, S190A	T19N, A169S	E75D

48_S23 (ST63)	64	X	Catheter tip (neuronal)	–			

14_S7 (ST62)	32	X	Sputum (ICU)	–	NM	NM	NM

***E. coli*^§^**							

10_S4 (ST167)	256	X	Urine (outpatient)	–	Deletions of WVF at positions 77–79, T117I, D187G	G66D, V93A, A174E	Q212E

***K. michiganensis*^¶^**							

69_S35 (ST170)	256	X	Urine (outpatient)	*oqxB, oqxA*	M176V	N100D, D112E	Y53H

^†^ *C. freundii* ATCC 8090 = MTCC 1658 (PRJNA177199) was used as a reference strain for the comparative genomics to find amino acid mutations.

^‡^MLST unknown.

^§^ *E. coli ATCC 25922* (CP009072) was used as a subject in the comparative genomics analysis to find the amino acid mutations.

^¶^ *K. michiganensis* KCTC 1686 (CP003218.1) was used as a reference strain for this comparative genomic analysis to find amino acid mutations.

ICU: Intensive care unit; MLST: Multi-locus sequence typing; NM: No mutation.

To obtain true mutations from evolutionary modifications, only mutations that occurred in conserved regions in only resistant strains were tabulated and included as potential NFT-R-mediating mutations. This was done by aligning all the nfsA, nfsB and ribE amino acids from the respective genomes per species using BioEdit [24] and finding the mutations in conserved areas.

### Phylogenomic analysis of nitrofurantoin resistant strains

**Figure 1.**
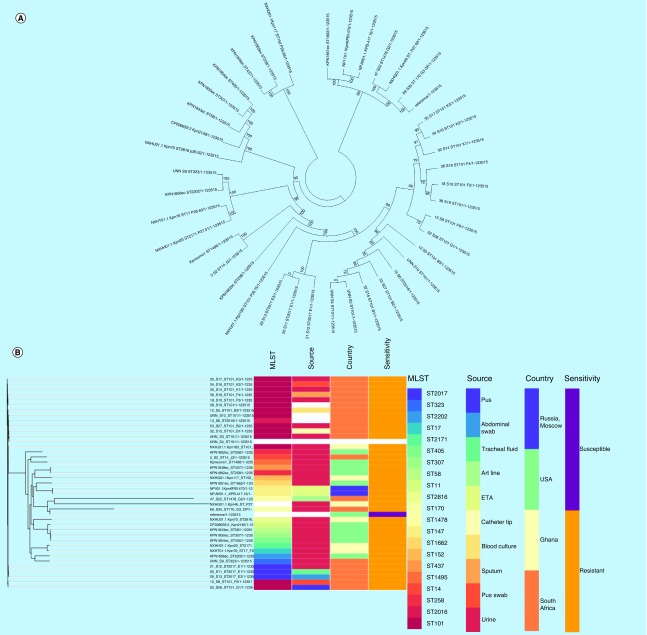
**Evolutionary relationship of nitrofurantoin-resistant *Klebsiella pneumoniae* strains from different parts of the world.** Phylogenomic tree of **(A)** *Klebsiella pneumoniae* drawn with MEGA 7 and **(B)** *K. pneumoniae* and associated metadata drawn with Phandango. The isolates clustered according to clones and country of origin, although the genomic phylogeny shows a closer clustering of strains of different clones and countries. Strains of ST101 were not all of the same clade while many strains from the same hospitals and wards were of the same clade.

**Figure 2.**
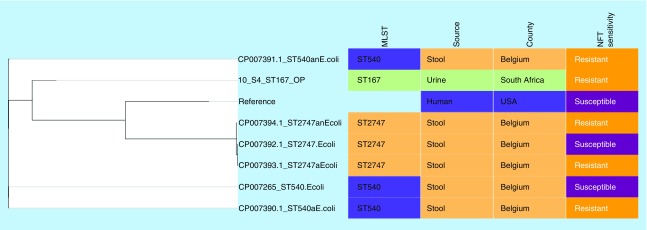
**Phylogenomic tree of *Escherichia coli* nitrofurantoin-resistant strains from Belgium, USA and South Africa.** Clustering of the strains into clades were mainly country- and clone-specific. MLST: Multi-locus sequence typing; NFT: Nitrofurantoin.

**Figure 3.**
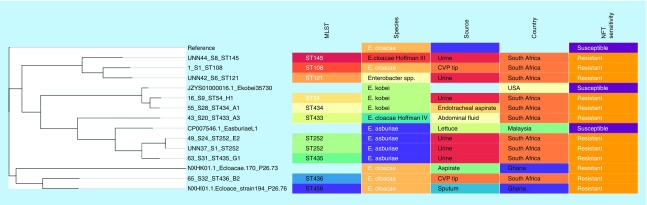
**Phylogenomic tree of nitrofurantoin resistance *Enterobacter species* from South Africa, USA, Malaysia and Ghana.** Clustering of the strains into clades were mainly country- and clone-specific except for 65_S32 of ST436 from South Africa and NXHI01.1 of ST455 (Ghana), and between 1_S1 of ST108 and the *Enterobacter* spp. strain UNN42_S6. MLST: Multi-locus sequence typing; NFT: Nitrofurantoin.

**Figure 4.**
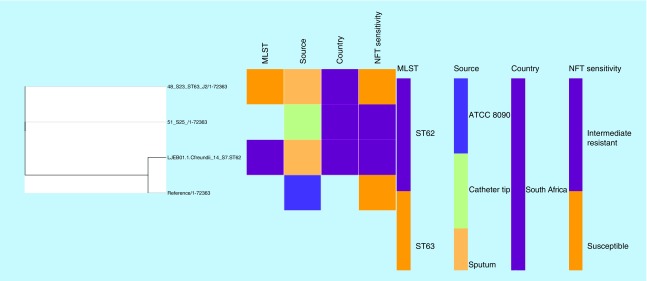
**Phylogenomic tree of *Citrobacter freundii* nitrofurantoin intermediate resistant strains from South Africa.** Clustering of the strains into clades were mainly country- and clone-specific. MLST: Multi-locus sequence typing; NFT: Nitrofurantoin.

Genomes of NFT-R *K. pneumoniae* (from bioproject PRJNA287968, PRJNA411997 and PRJNA376414)*, E. coli* (accession numbers CP007265.1, CP007390.1, CP007391.1, CP007392.1, CP007393.1, CP007394.1) and *Enterobacter* (JYS01000016.1, CP007546.1, NXHK01000000, NXHI01000000) strains as well as those of susceptible and reference strains were searched for and downloaded from PATRIC and NCBI (Genbank). These genomes, in addition to those included in this study, were used to draw a global phylogenetic tree of NFT-R isolates using the CSI phylogeny server [25] and Parsnp v1.2 [26]; the parsnp command line was executed with ‘-C 1000’ flag to force alignment across large collinear regions. Parsnp phylogenies were viewed with Gingr [27]. The obtained phylogenomic trees were downloaded in Newick format and annotated or edited with Figtree 1.4.3 [28] and/or MEGA 7 [29]. The edited trees were coupled with their metadata using Phandango (Figures 1–4) [30].

## Results & discussion

### Sources & types of specimens, nitrofurantoin resistance rates & levels

The MICs of NFT for all the isolates ranged from 32 to 512 mg/l, which makes most (n = 31) of the isolates resistant per the Clinical Laboratory Standards Institute (2016) breakpoints: resistance > 64 mg/l (Tables 1–3) [31]. The MICs of *K. pneumoniae/michiganensis, Enterobacter* spp. *and E. coli* were mostly ≥128 mg/l, an indication of the high-level NFT resistance in these isolates. Of note, 86.1% (31/36) of the isolates were resistant, 11.1% (4/36) were intermediates and 2.7% (1/36) were susceptible. Two of the *C. freundii* isolates (n = 3) were intermediate resistant (Table 3). This study confirmed the fact that NFT resistance is mostly pathogen-specific [32] as it was potent against *Citrobacter* spp., but *K. pneumoniae/michiganensis, E. coli* and *Enterobacter* spp. were largely resistant, corroborating earlier findings [33]. The NFT resistance observed in the isolates in addition to their already reported resistance to penicillins [18], cephalosporins [18], carbapenems [18], fluoroquinolones [17], colistin and tigecycline [5] makes most of the isolates MDR and pandrug-resistant.

Uropathogens isolated from UTI patients in South Africa basically include *E. coli, K. pneumoniae* and *Enterococcus faecalis* with very high susceptibility to NFT (91.7−94%) [34,35], albeit fewer *E. coli* isolated from urine and very high NFT-R is reported herein. As well, the presence of extended-spectrum β-lactamases (ESBLs) and carbapenemases in uropathogens isolated from South Africa has been minimal while this study's isolates had several ESBLs and carbapenemases [5,17,18]. The MDR nature of these strains [5,17,18] will be a great challenge to clinicians and threat to public health should they spread to other hospitals. Particularly, high NFT-R (83.3–83.9%) was detected among *E. coli* isolated from water and sediments collected from the Apies river in Gauteng, South Africa, suggesting that the water bodies are being polluted with NFT or NFT-R isolates [36]. Therefore, a comprehensive surveillance of NFT-R and NFT stewardship is necessary to prevent further reports of NFT-R in both patients and the environment.

To my knowledge, such high-level NFT-R among several clinical Enterobacteriaceae species have not been reported worldwide and studies reporting on NFT-R rates have shown that NFT-R among uropathogens remains low (1.3–4%), with a higher resistance rate only being recorded in non-OECD (Organization for Economic Cooperation and Development) countries (17%) [37,38]. As well, the high resistance rates of NFT observed in these isolates is worrying, particularly when it has been shown that resistance to NFT develops slowly and rarely [39]. In addition, the presence of such high-level NFT-R among these isolates suggest that there could be a higher level and prevalence of NFT-R in Durban compared with the lower rates reported in Gauteng (Pretoria) [34,35], and that a comprehensive surveillance is needed to inform antibiotic treatment guidelines for UTIs [9,16].

The isolates were obtained basically from eight sources viz., urine (n = 17), central venous puncture tip (n = 2), sputum (n = 3), pus (n = 3), catheter tip (n = 2), endotracheal aspirate (n = 1), abdominal fluid/swab (n = 1) and arterial line (n = 1). The three *C. freundii* isolates were from sputum (n = 2) and central venous puncture tip (n = 1) while the two susceptible *K. pneumoniae* strains were from urine. Thus, most of the susceptible isolates were from sputum and urine, in other words, noninvasive devices and procedures. Furthermore, the presence of NFT-resistant Enterobacteriaceae in most urine samples (n = 17) is worrying as NFT will be unable to clear these strains.

Given the importance of NFT in acute uncomplicated UTIs [16,39] and its renaissance/revival in combination therapies to manage MDR infections [6,9], increased care should be taken in the prescription of NFT to avoid further escalation of NFT resistance among Enterobacteriaceae. The stewardship of NFT will prolong its usefulness for uncomplicated UTIs, particularly when NFT has been found to be more efficacious for UTIs with mild side effects [39]. Moreover, species identification and antibiotic sensitivity testing of pathogens implicated in UTIs are necessary to avoid prescribing NFT for *Serratia marcescens* or NFT-resistant uropathogens respectively, as most *S. marcescens* are intrinsically resistant to NFT [33,40]. As such, empirical NFT prescription for UTIs should be done with caution as it could fail in patients with NFT-resistant strains (including *Salmonella* spp.*, Proteus* spp. and *Pseudomonas* spp., most of which are intrinsically resistant) [33].

The higher NFT-R rate among these carbapenem-resistant Enterobacteriaceae (CRE) isolates provides useful insights into the level of drug resistance in hospitals (in Durban), specifically surgical wards (n = 5) and intensive care units (ICUs) (n = 20) from which these strains were largely taken from. It also substantiates the association of MDR bacteria, including CRE, with invasive medical instruments/procedures (n = 10), surgical units and ICUs (Tables 1–3) [41–43]. While it is unfortunate that NFT was not effective against these CRE as reported in other studies [9,11], it is an indication of the challenges facing clinicians presented with MDR infections; specifically, restricted antibiotic options. Moreover, it underscores the need to undertake periodic surveillance of hospitals and invasive medical devices, as well as moderate the use of invasive procedures to prevent reinfection of patients [41]. Such surveillance should not involve the use of rectal swabs as the low-level concentrations of NFT in the rectum have been cited as a reason for the higher NFT-R recorded among intestinal bacteria [44,45].

### Effect of efflux on nitrofurantoin resistance

**Figure 5.**
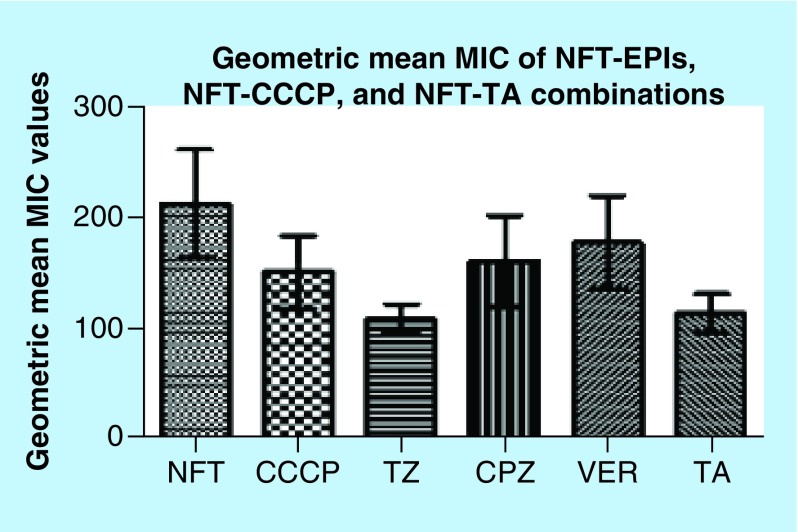
**Geometric mean of nitrofurantoin, nitrofurantoin-carbonyl cyanide m-hydrophenylhydrazine, nitrofurantoin-tannic acid and nitrofurantoin-efflux pump inhibitor minimum inhibitory concentrations.** TZ and TA significantly reduced the geometric mean MIC of NFT (p < 0.001) than the remaining efflux-inhibiting agents. CCCP: Carbonyl cyanide m-hydrophenylhydrazine; CPZ: Chlorpromazine; EPI: Efflux pump inhibitor; MIC: Minimum inhibitory concntration; NFT: Nitrofurantoin; TA: Tannic acid; TZ: Thioridazine; VER: Verapamil.

The MICs of NFT, efflux pump inhibitors (EPIs), carbonyl cyanide m-hydrophenylhydrazine (CCCP) and tannic acid (TA) alone, and of NFT in combination with EPIs, CCCP and TA for the isolates are shown in Supplementary Tables 1 & 2. Interestingly, the inhibitors did not have a drastic effect on the MICs of NFT. In other words, none of the inhibitors had a fold change of >4; MIC fold changes of 1–2 were recorded for the selected species/clones except three strains that had a fold change of 4 (Supplementary Table 1). None of the inhibitor- or TA-NFT combinations resulted in any MIC fold change in *C. freundii* isolates. The EPIs, CCCP and TA used in combination with NFT are known to directly or indirectly block/inhibit the activity of specific efflux pumps families in bacteria [5,17]. Phenyl arginine β-naphthylamide (PaβN), which is known to block resistance-nodulation division (RND) efflux pumps in Gram-negative bacteria, specifically *Pseudomonas aeruginosa* [46], also failed to change the MICs of NFT on a randomly sampled number of isolates (data not shown). Thioridazine (TZ) and TA had a significant effect on the geometric mean MIC of NFT (p < 0.001) (Supplementary Table 3) by respectively reducing it from 212.92 to 109.53 and 114.46. The geometric mean MIC values of the EPI-NFT, CCCP-NFT and TA-NFT combinations are depicted in Figure 5.

The inability of any of these EPIs to reduce the MICs of NFT significantly or reverse NFT-R indicates the little or no role played by efflux in NFT-R mechanisms, which is contrary to what has been already reported in Hong Kong in which *oqxAB* was implicated in NFT-R [16]. Furthermore, the results suggest that efflux pumps are involved in NFT resistance to different degrees in different Enterobacteriaceae species and clones. Thus, efflux is not a major or significant NFT-R mechanism and blocking efflux pumps cannot reverse NFT-R in Enterobacteriaceae.

### *oqxAB* efflux gene is a complementary nitrofurantoin resistance mechanism

Annotation of the genomic data identified *oqxAB*, which has been reported to be important in NFT resistance (Tables 1–3 & Supplementary Table 4) [16]. *oqxAB* was found in 31 isolates (Supplementary Table 3), and was found in the chromosomes of *K. pneumoniae* ST101 and ST2017. *oqxA* was present in 20 isolates while *oqxB* occurred in 32. The frequency of *oqxA/B* per species are shown in Supplementary Table 3. There was no *oqxAB* in *C. freundii*, which were either susceptible (n = 1) or intermediate resistant (n = 2). Interestingly, *oqxB* was present in most NFT-R (41/43) and NFT-I (2/4) isolates comprising of almost all the species, a situation also observed by Ho *et al.* (2016) [16].

There was no strong association between minor but clinically relevant MIC fold changes (Δ = 1–4) observed with NFT-inhibitor combinations and *oqxAB* [47]. For instance, *E. coli* (10_S4) had a clinically relevant decrease in NFT MIC from 256 to 128 mg/l upon adding TZ and TA, but there was no *oqxAB* present in this organism. Furthermore, *oqxB* was present in all *K. pneumoniae* ST101, ST2016 and ST2017 strains while *oqxA* was present in only six (53_S27, 52_S26, 21_S12, 20_S11, D and C) strains. Yet, higher MICs (256–512 mg/l) were recorded in strains without *oqxA* than in strains with both genes (128 mg/l) in all but three strains (256 mg/l) (Table 1). Further studies will be necessary to investigate the functional independence of *oqxB* from *oqxA* in increasing NFT MICs in Enterobacteriaceae, specifically *K. pneumoniae*.

Based also on the insignificant MIC fold changes observed after adding the inhibitors to NFT, it can be suggested that *oqxAB* genes are a complementary NFT-R mechanism that is adding up to nfsA, nfsB and ribE mutations to yield high-level NFT resistance in the strains [13].

Specific plasmids or replicon/incompatibility types associated with the *oqxAB* genes could not be predicted due to the truncated nature of the plasmid contigs. Moreover, there were no IS26 elements or transposons in the immediate environment of the *oqxAB* genes as has been reported elsewhere [16]. Therefore, the spread of NFT resistance cannot be attributed substantially to horizontal gene transfer through mobile genetic elements but through clonal and polyclonal expansion of the Enterobacteriaceae strains.

### Mutations in *nfsA*, *nfsB* & *ribE* collectively confer high-level resistance to nitrofurantoin

Alignment of *nfsA*, *nfsB* and *ribE* sequences in wild-type strains (specified in the methods) against those of this study was undertaken to determine the presence of mutations that could account for the high-level NFT-R. Tables 1–3 show the unique mutations recorded in the conserved regions of these three proteins in at least *E. coli* and *K. pneumoniae* isolates, which could collectively account for the high-level NFT resistance recorded in the strains. Of note, mutations in these chromosomal genes (*nfsA, nfsB* and *ribE*) or proteins mostly reflected the clonal relatedness of the strains, in other words, mutations were mostly species and clone specific (Figures 1–3). Of the six clones in *K. pneumoniae*, three (ST101, ST2016 and ST2017) had the same mutations in nfsA (R203C), nfsB (R207L) and ribE (R58H, D203E) whose combined effect could be responsible for the high-level NFT-R (128–512 mg/l) in these clones. Moreover, the differences in MICs (128–512 mg/l) among these clones having *oqxAB* genes and the same nfsA, nfsB and ribE mutations suggest that other unknown NFT-R mechanisms might be at play in these strains as suggested by Ho *et al.* (2016) [16]. The mutation in nfsA (R203C) found in these three clones is similar to that reported to cause NFT-R in *E. coli* (R203C/L) by Sandegren *et al.* (2008) [48].

*K. pneumoniae* ST14 and ST323 also had the same mutation in ribE (D203E) with no mutation(s) in both nfsA and nfsB. These two clones had an intermediate resistance to NFT (64 mg/l), suggesting that the D203E mutation in ribE could cause low-level NFT resistance; further complementation and mutagenesis studies will be necessary to confirm this. There were unique mutations in *K. pneumoniae* ST1478 that also need confirmation through mutagenesis/transcomplementation studies.

With respect to *Enterobacter* spp., all the nine different clones and species (*E. cloacae, E. asburiae, E. kobei, E. cloacae Hoffman complexes III* and *IV* and *Enterobacter* spp.) exhibited unique mutations as well as same mutations (Table 2). For instance, five clones (ST434, ST54, ST108, ST145 and ST121) shared the same mutations in nfsA (M201L, G204D) and ribE (V42I). However, these five clones further differed with respect to their mutations in nfsB: *E. kobei* (ST434 and ST54) had the same mutations in nfsB (A19T, D25E, A155G, L157M and L186V) while *E. cloacae* (ST108), *E. cloacae complex ‘Hoffman cluster III’* (ST145) and *Enterobacter* spp. (ST121) also shared the same mutations (D25E, N109A, T122C, L157M) in nfsB. *E. asburiae* (isolates 49_S24 and A(UNN37_S1)) clones ST252 (n = 2) had the same mutations in all the three genes analyzed. The species-specific and clonal-specific nature of the mutations suggest that the *Enterobacter* spp. were either exposed to different environments (aerobic or anaerobic) with varying NFT selection pressures or mutated according to clonal/species serendipities [44,45].

*C. freundii* strains 51_S25 and 48_S23 (ST63) had the same mutations in all three genes analyzed, including an insertion of LGLAEQLLLGVVDTAMI between positions 94 and 95. Hence, it is not surprising that they had the same NFT MIC (64 mg/l), suggesting that the mutations and InDels found in these two isolates collectively mediate intermediate NFT resistance; as well, further mutagenesis studies may confirm or show otherwise as a single or a few of these mutation(s) might actually be responsible for this intermediate NFT resistance. *C. freundii* ST62, which was susceptible to NFT (32 mg/l), had no mutation(s) in nfsA, nfsB and ribE proteins, explaining the observed NFT susceptibility in this clone.

*E. coli* remains the most studied species to date among all the organisms included in this study with regards to NFT-R mechanisms. Interestingly, for *E. coli* ST167 (10_S4), T117I and D187G mutations in nfsA (deletions of WVF at positions 77–79 were found in this study's strain while W77*stop was detected by Sandegren *et al.* [2008]), and G66D in nfsB has already been reported in different clinical clones (ST540 and ST2747) from Europe and Canada, as responsible for high-level resistance to NFT *in vitro* [44,48]. This confirms the resistance mechanism underlying NFT resistance in this *E. coli* isolate and suggests that this mutation is common among *E. coli* strains of different clonality and geographical sources. *K. michiganensis* also had unique mutations in all the three chromosomal genes (Table 3).

### Evolutionary & phylogenomic relationship of nitrofurantoin-resistant strains

Global phylogenomic analysis using NFT-R *K. pneumoniae, E. coli, E. cloacae* and *C. freundii* genomes from NCBI and PATRIC, together with those of the strains used in this study are shown in Figures 1–4, respectively. The *K. pneumoniae* NFT-R strains whose genomes were included in this study were mainly from the USA (TX), Russia (Moscow), Ghana (Kumasi) and South Africa (Pretoria) as well as from diverse samples and different clones (Figure 1A & B). It is evident from the phylogenomic tree in Figure 1A & B that the resistant strains mainly clustered according to clones and country of origin within a clade, with a few exceptions in which strains from South Africa and Ghana clustered with those from the USA. For instance, KPN1856ec of ST2202 from USA and UNN_S9 of ST323 from South Africa were of the same clade. The same clustering was observed between Kpneumo1 of ST1496 from USA and 3_S2 of ST14 from South Africa, as well as between 47_S22 (ST1478, South Africa) and NXHG01.1KpN46 (Ghana). Among the *E. cloacae* strains, 65_S32 of ST436 from South Africa and NXHI01.1 of ST455 (Ghana) belonged to the same clade while 1_S1 of ST108 and the *Enterobacter* spp. strain UNN42_S6 were closely related to each other, albeit they were of different species (Figure 3).

The *E. coli, E. cloacae* and *C. freundii* phylogenomic trees, like that of *K. pneumoniae*, show that NFT resistance was mainly of local emergence than by international transmission. This is obviously due to the nonplasmid mediated dissemination of NFT resistance among Enterobacteriaceae, which limits the spread of NFT resistance to clonal and multiclonal expansion. Thus, it can be argued that NFT resistance in South Africa emerged locally, followed by clonal expansion through same and different hospitals. However, the closer evolutionary association between different clones from different countries as shown above (Figures 1 & 3) suggests the potential for international transmission of NFT-R strains.

These figures also show the higher resolution of whole genome sequencing over MLST typing as strains belonging to the same clone (or sequence type; ST) were closely related to strains from different ST. Moreover, it is easier to identify the species and strains of an isolate using whole genome sequencing phylogeny by using the clustering or evolutionary distance between that isolate and closely related species and strains on the tree. In particular, Figure 3 shows that the *Enterobacter* spp. isolate has a very close evolutionary relationship to *E. cloacae* than to other species of *Enterobacter*. *K. pneumoniae* strains of ST101 were all not of the same clade as expected, but rather clustered into separate clades that largely reflected the hospital and wards from which the strains were isolated from, indicating the intra- and inter-hospital spread of NFT resistance in clonal and polyclonal Enterobacteriaceae in Durban. These evidences further support the need for genomic epidemiology of infectious diseases as a more effective tool for tracing the spread of infections as nongenomic typing methods such as MLST could be misleading.

## Conclusion & limitations

In conclusion, diverse mutations in the nfsA, nfsB and ribE proteins, complemented by o*qxAB* efflux genes, are responsible for the high-level NFT-R in clinical Enterobacteriaceae in Durban, South Africa. NFT-R emerged locally or independently in South Africa and other parts of the world and is being spread vertically through clonal and multiclonal expansion within and between wards and hospitals. Furthermore, this high-level NFT-R is associated with surgical wards and ICUs as well as with invasive medical devices/procedures and urine, which warrants a comprehensive surveillance of hospitals to inform antibiotic choices for uncomplicated UTIs. But for this study, NFT was known to have little prevalence and low levels of resistance among uropathogens [9,14,39]. This finding of high-level NFT resistance is worrying as it is being increasingly used in combination therapy to manage infections that are resistant to last-resort antibiotics (carbapenems, colistin and tigecycline).

The study was limited by the absence of transcomplementation studies to ascertain the MIC effect of the identified mutations. It was also limited by the noninclusion of several susceptible *E. cloacae* parental or wild-type strains per ST/clones and species to show that the amino acid sequences of the nfsA, nfsB and ribE proteins were conserved within susceptible strains. Nevertheless, inclusion of the genomic data of other NFT-susceptible isolates from GenBank made up for this limitation.

## Future perspective

Decreasing costs in whole-genome sequencing coupled with increasing bioinformatics skills and open-source software for analyzing bacterial genomes will increase genomics-based antibiotic resistance research outputs. This is welcoming, as genome-based analysis of resistance determinants and molecular epidemiology through phylogenomics provide a higher resolution than PCR and PCR-based phylogenetics. Evidently, such genome-wide association studies shall provide detailed insights into the causes and dissemination routes of antibiotic-resistant infections. While NFT resistance remains low among enterobacteria, it is expected to rise as its use is increased to cater for increasing MDR among uropathogens. Furthermore, detailed transcriptional profiling using RNAseq, CRISPR-Cas9 and functional studies are necessary to identify other unknown and evolving NFT resistance mechanisms. Thus, it is needful to increase surveillance and monitoring of NFT resistance globally to promptly advise on resistance outbreaks, stewardship and treatment guidelines.

**Summary points**

- Due to the importance of nitrofurantoin in the treatment of uncomplicated urinary tract infections, it is becoming important in carbapenem-resistant Enterobacteriaceae (CRE) infections of the urinary tract; especially when resistance to nitrofurantoin develops slowly and scarcely.
- There is high-level nitrofurantoin resistance in private hospitals in Durban, South Africa, which is unprecedented worldwide; particularly in CRE.
- The detection of high-level nitrofurantoin resistance among CRE that are also extra drug resistant makes this a public health threat worthy of attention.
- Notably, most of these extradrug- and pandrug-resistant isolates were isolated from urine, invasive devices, intensive care units, etc. and were of the same clones and species from same and different wards of various private hospitals in Durban.
- Diverse resistance-conferring mutations in *nsA, nfsB* and *ribE* were identified in specific clones and species, showing the independent emergence and subsequent vertical transfer of these extradrug- and pandrug-resistant strains.
- Efflux was not a major mechanism of nitrofurantoin resistance, contrary to already published data showing the importance of *oqxAB* in nitrofurantoin resistance.
- Nitrofurantoin resistance expansion in Enterobacteriaceae were clonal and multiclonal, per the MLST data obtained from the genomes.
- Further phylogenomic analysis of nitrofurantoin-resistant Enterobacteriaceae from various parts of the world showed the independent and local emergence of nitrofurantoin resistance among Enterobacteriaceae globally.
