# Supplementary material for: Genomic insights into nitrofurantoin resistance mechanisms and epidemiology in clinical Enterobacteriaceae
Source: Future Sci OA. 2018 Feb 27;4(5):FSO293. doi: 10.4155/fsoa-2017-0156 (PMC5961450; doi:10.4155/fsoa-2017-0156)
Supplement: Supplementary file 1 [file fsoa-04-293-s1.docx]

**Genomic Insights into Nitrofurantoin Resistance Mechanisms and Epidemiology in Clinical Enterobacteriaceae.**

1. **Materials and Methods**
   1. **Bacterial strains, sensitivity testing, and efflux inhibition assays.**

Thirty-six clinical CRE strains collected between 2012-2013 with already sequenced and published genomes were used for the phenotypic and genomic characterisation of NFT resistance mechanisms [1–3]. These strains comprised of *Klebsiella pneumoniae*(n=21)*, Enterobacter spp.*(n=10)*, Citrobacter freundii*(n=3)*, Escherichia coli*(n=1), and *Klebsiella michiganensis*(n=1), which have been already reported to have resistance to carbapenems, tigecycline, colistin, and fluoroquinolones [1–3]. The *Enterobacter spp.* were composed of *Enterobacter cloacae* (n=2), *Enterobacter asburiae* (n=3), *Enterobacter kobei* (n=2), *Enterobacter cloacae complex “Hoffman cluster IV”* (n=1), *Enterobacter cloacae complex “Hoffman cluster III”* (n=1), and *Enterobacter spp.* (n=1). The *K. pneumoniae* strains were of multi-locus sequence type (MLST) ST101 (n=14), ST2017 (n=3), ST2016 (n=1), ST323 (n=1), ST14 (n=1), and ST1478 (n=1). There were singletons of *E. coli* ST167, *K. michiganensis/oxytoca* ST170, and *C. freundii* ST62 and ST3. Besides *E. cloacae* ST252 (n=2), all other *E. cloacae* clones were singletons: ST54, ST108, ST121, ST145, ST434, ST435, and ST436 [2,3,1]. Most of these strains were isolated from surgical wards (n=5), neuronal wards (n=3) and intensive care units (ICUs) (n=20) (Table 1).

Micro-broth dilution was used to screen the isolates to identify their NFT MICs using already established methods [4]. The MIC results were interpreted per CLSI 2016 breakpoints (Table 1) [5]. MICs of efflux-pump inhibitors (EPIs) including verapamil (VRP), thioridazine (TZ) and chlorpromazine (CPZ) as well as tannic acid (TA) and the protonophore carbonyl cyanide m-hydrophenylhydrazine (CCCP) were determined using already described methods [4]; as well, the EPI Phenyl Arginine β-naphthylamide (PaβN) was used on a random number (n=8) of isolates to assess its effects (data not shown) [2,3]. This was done to ascertain the activity of these EPIs alone on the isolates and to select appropriate sub-MICs for combination with NFT. The sources and methods of preparing solutions of these EPIs, TA, and CCCP have been described elsewhere [2,3]. The two-dimensional checkerboard method was used to determine the effect of increasing concentrations (which are sub-MICs) of EPIs, TA, and CCCP on the MICs of NFT on 22 selected isolates representative of every clone and species in the 36 isolates (Table S1) [2,3,6]. The concentrations of EPIs, CCCP and TA are used with NFT are shown in Table S2. *E. coli* ATCC 25922 (ST73) and *K. oxytoca* ATCC 13178 were used as controls in the antibiotic sensitivity testing (Table S1). All MIC assays were carried out in triplicates.

- 1. **Data and statistical analyses**

NFT MIC fold changes ($\Delta$) upon addition of sub-MIC of EPIs, TA, and CCCP were calculated with this formula: (*MIC of NFT alone) / (MIC of NFT+EPI, TA or CCCP).* A $\Delta$ of ≥4 was accepted as significant and a p-value of <0.05 was defined as statistically significant. Geometric mean MIC of NFT and NFT-EPIs, NFT-CCCP, and NFT-TA were calculated as already described [7]. GraphPad Prism 5.0 for Windows (GraphPad Software, San Diego, CA, USA) was used for all the statistical analyses to determine the significance of the NFT $\Delta$ in the presence of EPIs, TA, and CCCP (Tables S1). This was carried out using non-parametric one-way analysis of variance (ANOVA) followed by Dunnett’s multiple comparison test.

**References**

1. Osei Sekyere J, Govinden U, Essack SY. Comparison of Existing Phenotypic and Genotypic Tests for the Detection of NDM and GES Carbapenemase- Producing Enterobacteriaceae. *J Pure Appl Microbio*. 10(4), 2585–2591 (2016).

2. Osei Sekyere J, Amoako DG. Carbonyl Cyanide m-Chlorophenylhydrazine (CCCP) Reverses Resistance to Colistin, but Not to Carbapenems and Tigecycline in Multidrug-Resistant Enterobacteriaceae. *Front. Microbiol.* [Internet]. 8(February) (2017). Available from: http://journal.frontiersin.org/article/10.3389/fmicb.2017.00228/full.

3. Osei Sekyere J, Amoako DG. Genomic and Phenotypic Characterisation of Fluoroquinolone Resistance Mechanisms in Enterobacteriaceae in Durban, South Africa. *PLoS One*. accepted(In Press), 1–14 (2017).

4. Clinical and Laboratory Standards Institute (CLSI). Methods for Dilution Antimicrobial Susceptibility Tests for Bacteria That Grow Aerobically; Approved Standard--Tenth Edition. Broth Microdilution Method. , 17–20 (2015).

5. Clinical and Laboratory Standards Institute (CLSI). Performance standards for Antimicrobial Susceptibility Testing; Twenty-Seventh Informational Supplement M100-S27. CLSI, Wayne, PA, USA, USA.

6. King AM, Reid-Yu SA, Wang W, *et al.* Aspergillomarasmine A overcomes metallo-β-lactamase antibiotic resistance. *Nature* [Internet]. 510(7506), 503–6 (2014). Available from: http://www.ncbi.nlm.nih.gov/pubmed/24965651.

7. Ho P, Ng K, Lo W, *et al.* Plasmid-Mediated OqxAB Is an Important Mechanism for Nitrofurantoin Resistance in Escherichia coli. 60(1), 537–543 (2016).
