# Supplementary material for: Genomic insights into nitrofurantoin resistance mechanisms and epidemiology in clinical Enterobacteriaceae
Source: Future Sci OA. 2018 Feb 27;4(5):FSO293. doi: 10.4155/fsoa-2017-0156 (PMC5961450; doi:10.4155/fsoa-2017-0156)
Supplement: Supplementary file 2 [file fsoa-04-293-s2.docx]

**Genomic Insights into Nitrofurantoin Resistance Mechanisms in Clinical Enterobacteriaceae.**

***Table S1*: Nitrofurantoin (NFT) MIC fold changes on selected enterobacterial clones upon adding Carbonyl Cyanide-m-Chlorophenylhydrazine (CCCP), thioridazine (TZ), chlorpromazine (CPZ), verapamil (VRP), and tannic acid (TA).**

| Isolate | MLST TYPE | MIC of Nitrofurantoin (NFT) (mg/L)^[[1]](#footnote-1)^ | | | | | |
| --- | --- | --- | --- | --- | --- | --- | --- |
|  |  | NFT | NFT + CCCP [8-128] ($\Delta$) | NFT + TZ [64-256] ($\Delta$) | NFT + CPZ [16-256] ($\Delta$) | NFT + VRP [256] ($\Delta$) | NFT + TA [256] ($\Delta$) |
| *E. coli ATCC 25922* | ST73 | **16** | 16 (1) | 16 (1) | 16 (1) | 16 (1) | 16 (1) |
| *K. oxytoca ATCC 13178* | -^1^ | **16** | 16 (1) | 16 (1) | 16 (1) | 16 (1) | 16 (1) |
| *Klebsiella pneumoniae* |  |  |  |  |  |  |  |
| C(UNN39_S3) | ST101 | **256** | 256 (1) | 128 (2) | 256 (1) | 256 (1) | 128 (2) |
| J(UNN46_S10) | ST101 | **512** | 256 (2) | 128 **(4)** | 512 (1) | 512 (1) | 256 (2) |
| I(UNN45_S9) | ST323 | **64** | 64 (1) | 64 (1) | 64 (1) | 64 (1) | 64 (1) |
| 3_S2 | ST14 | **64** | 64 (1) | 64 (1) | 64 (1) | 64 (1) | 64 (1) |
| 13_S6 | ST2016 | **256** | 256 (1) | 128 (2) | 256 (1) | 256 (1) | 128 (2) |
| 20_S11 | ST2017 | **256** | 256 (1) | 128 (2) | 256 (1) | 256 (1) | 128 (2) |
| 29_S13 | ST2017 | **256** | 128 (2) | 128 (2) | 256 (1) | 256 (1) | 128 (2) |
| 47_S22 | ST1478 | **128** | 128 (1) | 128 (1) | 128 (1) | 128 (1) | 128 (1) |
| *Enterobacter spp.* |  |  |  |  |  |  |  |
| A (UNN37_S1) | ST252 | **512** | 128 **(4)** | 128 **(4)** | 128 **(4)** | 256 (2) | 128 **(4)** |
| F (UNN42_S6) | ST121 | **256** | 256 (1) | 128 (2) | 128 (2) | 256 (1) | 128 (2) |
| H (UNN44_S8) | ST145 | **128** | 64 (2) | 64 (2) | 64 (2) | 128 (1) | 64 (2) |
| 1_S1 | ST108 | **256** | 128 (2) | 128 (2) | 128 (2) | 128 (2) | 128 (2) |
| 16_S9 | ST54 | **256** | 256 (1) | 128 (2) | 128 (2) | 128 (2) | 128 (2) |
| 43_S20 | ST433 | **256** | 256 (1) | 128 (2) | 256 (1) | 256 (1) | 128 (2) |
| 55_S28 | ST434 | **128** | 128 (1) | 64 (2) | 128 (1) | 128 (1) | 64 (2) |
| 63_S31 | ST435 | **128** | 128 (1) | 128 (1) | 128 (1) | 128 (1) | 128 (1) |
| 65_S32 | ST436 | **256** | 256 (1) | 128 (2) | 128 (2) | 256 (1) | 128 (2) |
| *E. coli* |  |  |  |  |  |  |  |
| 10_S4 | ST167 | **256** | 256 (1) | 128 (2) | 256 (1) | 256 (1) | 128 (2) |
| *Citrobacter freundii* |  |  |  |  |  |  |  |
| 14_ | ST62 | **32** | 32 (1) | 32 (1) | 32 (1) | 32 (1) | 32 (1) |
| 48_S23 | ST63 | **64** | 64 (1) | 64 (1) | 64 (1) | 64 (1) | 64 (1) |
| 51_ | -^1^ | **64** | 64 (1) | 64 (1) | 64 (1) | 64 (1) | 64 (1) |
| *Klebsiella michiganensis* |  |  |  |  |  |  |  |
| 69_S35 | ST170 | **256** | 128 (2) | 128 (2) | 128 (2) | 128 (2) | 128 (2) |

**Table S2: MICs of inhibitors and concentrations used for modulating NFT MICs**

| **Inhibitors** | **MICs (mg/L)** | **Inhibitor concentration used for modulating NFT MIC** |
| --- | --- | --- |
| CCCP | 16−256 | 8-128 |
| TZ | 128−≥512 | 64−256 |
| CPZ | 32−≥512 | 16−256 |
| VRP | >512 | 256 |
| TA | >512 | 256 |
| PaβN | 100 | 50 |

**Table S3: P-values of the Geometric mean MIC of NFT upon inhibition by CCCP, EPIs and TA**

| Antibiotic | Geometric Mean of MIC (mg/L) | | | | |  |
| --- | --- | --- | --- | --- | --- | --- |
|  | CCCP | TZ | CPZ | VRP | TA | PAβN |
| NFT−212.92 | 151.38* | 109.53*** | 161.23(ns) | 178.46(ns) | 114.46*** | 187.92(ns) |

**ns --> not significant, ∗P < 0.05, ∗∗P < 0.01 and ∗∗∗P < 0.001.**

***Table S4. Frequency and distribution of oqxAB efflux genes in the Enterobacteriaceae isolates. Adapted with permission from*** (Osei Sekyere and Amoako, 2017)

| Efflux genes | Gene variants | Frequency (N) of occurrence per specie | | | | | Total frequency |
| --- | --- | --- | --- | --- | --- | --- | --- |
|  |  | *K. pneumoniae* (n = 21) | *Enterobacter spp*. (n = 10) | *C. freundii* (n = 3) | *E. coli* (n = 1) | *K. michiganensis* (n = 1) | n=36 |
| *OqxAB* | *OqxA* | 9 | 10 | 0 | 0 | 1 | 20 |
|  | *OqxB* | 21 | 10 | 0 | 0 | 1 | 32 |

1. Unknown [↑](#footnote-ref-1)
